# Unilateral Concurrent Amyand’s Hernia and Inguinal Bladder Hernia (IBH): A Case Report

**DOI:** 10.7759/cureus.72640

**Published:** 2024-10-29

**Authors:** Nima Sadeghi, Jamie McDermott, Ayman Anasi, Brian Mayer, Imtiaz Ahmed

**Affiliations:** 1 Medicine, Midwestern University Arizona College of Osteopathic Medicine, Glendale, USA; 2 Radiology, Tempe St. Luke's Hospital, Tempe, USA

**Keywords:** amyand’s, amyand’s hernia, ibh, inguinal bladder hernia, inguinal hernia

## Abstract

An inguinal hernia is a common surgical condition where abdominal contents protrude through a weakened area of the abdominal wall. While most are straightforward, rare variants can lead to significant complications. Named after the surgeon who successfully removed a vermiform appendix from a hernia sac, Amyand's hernia is a rare finding. Similarly, an inguinal bladder hernia (IBH) is a rare condition where part of the urinary bladder protrudes into the inguinal canal. We present a unique case of a 62-year-old male who presented to the emergency department with a one-day history of lower abdominal pain, exacerbated by physical exertion. The absence of associated symptoms like nausea, vomiting, fever, or urinary symptoms made his condition challenging to diagnose clinically. However, computed tomography (CT) scans of the abdomen and pelvis confirmed the diagnosis of concurrent Amyand’s hernia and IBH. The definitive treatment for symptomatic and painful inguinal hernias involves surgery, either open or laparoscopic hernia repair, while asymptomatic, lower-risk inguinal hernias may be observed before intervention is warranted. This report underscores the challenge of balancing the benefits of standardizing diagnostic protocols, particularly preoperative diagnosis and management guidelines for rare variants of inguinal hernias, with the associated costs. In this case, the absence of typical urinary symptoms commonly seen in IBH further complicated the diagnostic process, exemplifying the challenge of identifying such rare anomalies. While standardized screening could help prevent serious and potentially fatal complications, it also poses a strain on medical resources and may lead to unnecessary emotional and physical stress for patients when screening for rare variants of inguinal hernias.

## Introduction

The term hernia refers to the protrusion of an organ or tissue through a weakened area or defect in the surrounding muscle or fascia that normally contains it. The presence of the vermiform appendix within an inguinal hernia sac was first described by Claudius Amyand, with the term “Amyand’s hernia” coined by Creese in 1953 [1]. Though rare, Amyand’s hernia may lead to inflammation, infection, or perforation [2,3]. Its reported incidence ranges from 0.19% to 1.7% of all hernia cases [4,5]. Preoperative diagnosis is often challenging, as Amyand’s hernia is typically an incidental finding during surgery [6]. Clinical exams, laboratory tests, and imaging frequently fail to differentiate it from other conditions [7]. Patients commonly present with sudden epigastric pain and localized tenderness in the right lower quadrant, often accompanied by a tender, irreducible inguinal mass. This presentation frequently mimics a strangulated hernia, making accurate preoperative diagnosis difficult.

A similarly rare variant of an inguinal hernia is an inguinal bladder hernia (IBH), which refers to a rare condition in which part of the urinary bladder herniates into the inguinal canal. Like Amyand’s hernia, IBHs are difficult to diagnose preoperatively, with only 7% identified before surgery [8]. This is largely because the hernias are often asymptomatic and may go undetected until discovered during surgery or incidentally on imaging performed for other reasons. Symptomatic patients may experience lower abdominal or inguinal pain along with urinary symptoms such as frequency, urgency, or incomplete voiding. In rare cases, bladder obstruction may occur, leading to more severe complications, including urinary retention, infection, or renal impairment.

Both Amyand’s hernia and IBHs are uncommon and pose diagnostic challenges due to their nonspecific symptoms, which often overlap with more typical hernia presentations. However, due to the potential for serious complications developing, prompt recognition of both an Amyand’s and IBH with appropriate preoperative imaging is essential as it can aid in planning for a modified surgical approach and lessen postoperative complications.

## Case presentation

**Figure 1:**
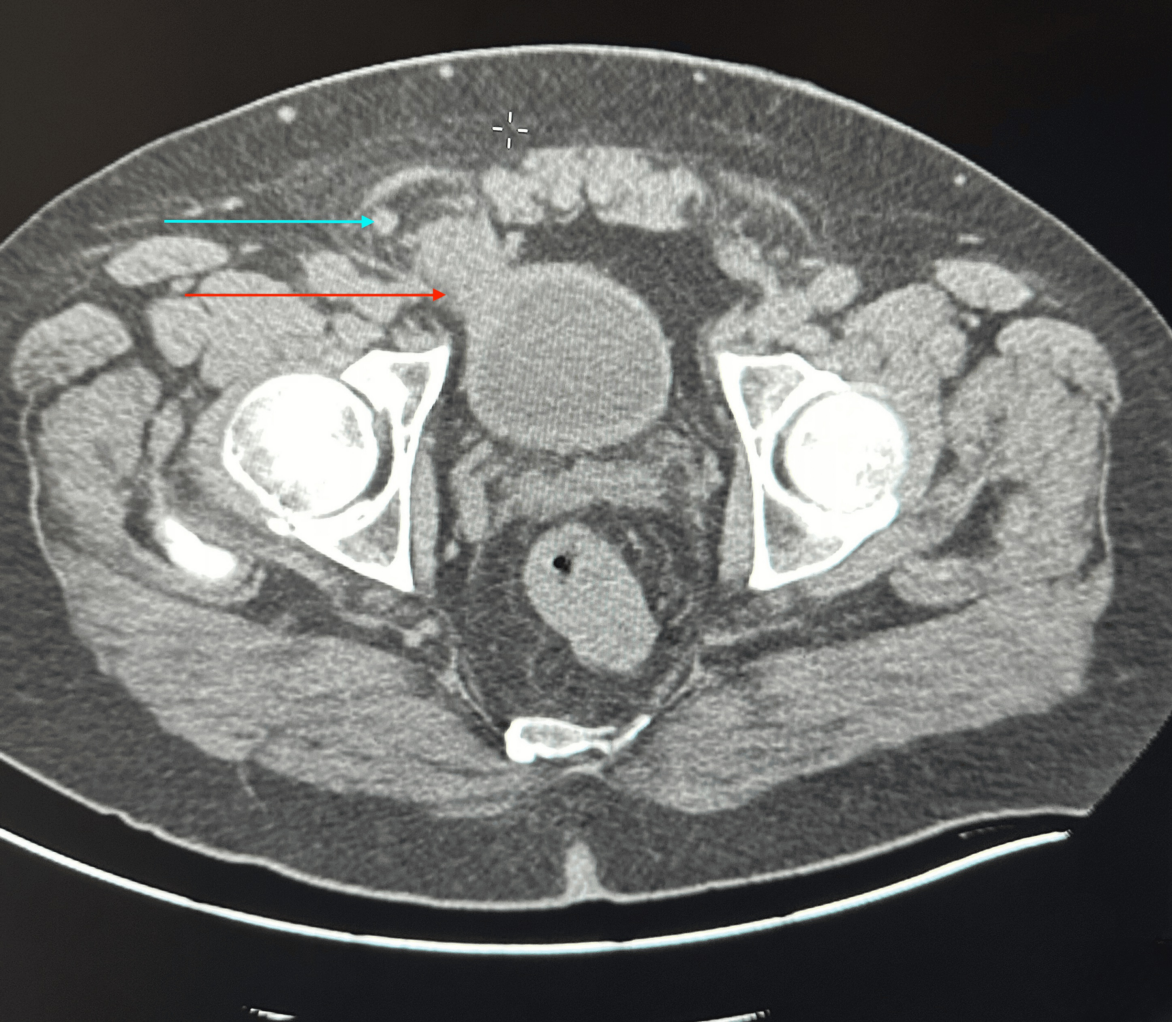
Axial CT scan depicting bladder protruding into the hernia sac with appendix encased Blue arrow indicates appendix; red arrow indicates bladder

**Figure 2:**
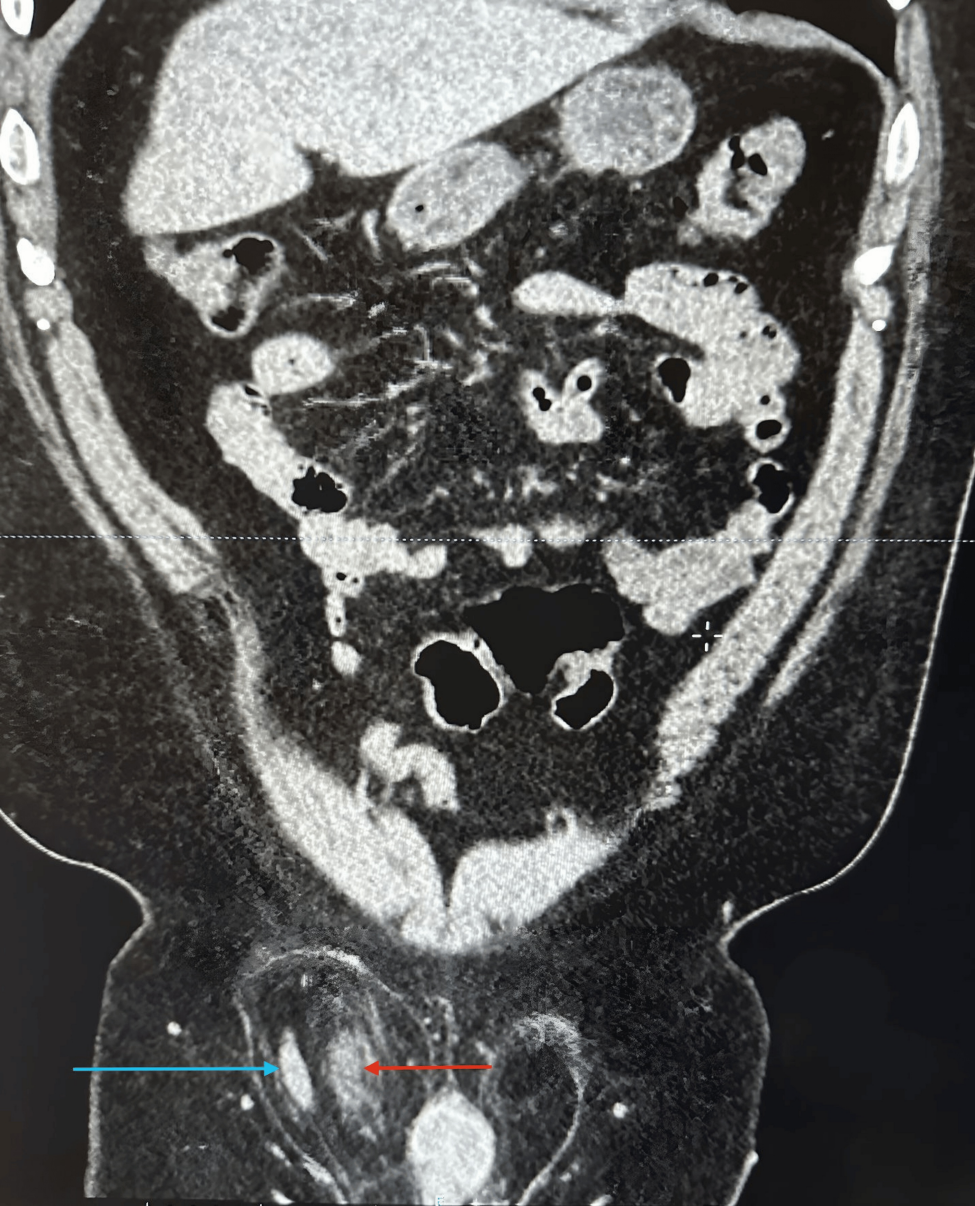
Coronal CT scan with presence of both Amyand's hernia and IBH Blue arrow indicates appendix; red arrow indicates bladder IBH: inguinal bladder hernia

We present a 62-year-old male patient who presented to the emergency department with a chief complaint of one-day lower abdominal pain that began one day prior and was exacerbated by physical activity. He did not endorse fever, nausea/vomiting, or other urinary symptoms. His past medical and surgical history was noncontributory. Laboratory tests were unremarkable, and a physical exam showed a non-reducible right inguinal mass with significant pain, particularly during the reduction maneuver. A computed tomography (CT) scan of the abdomen and pelvis with contrast was performed. CT imaging revealed herniation of the anterolateral wall of the bladder into the right inguinal canal along with herniation of the vermiform appendix into the inguinal sac, consistent with concurrent bladder herniation and Amyand’s hernia (Figures 1-2).

## Discussion

We present a 62-year-old male who visited the emergency department with a one-day history of acute onset lower abdominal pain, worsened by physical exertion. He denied associated symptoms such as fever, nausea, vomiting, or urinary complaints. After CT imaging of the abdomen and pelvis with contrast, the presence of concurrent Amyand’s hernia and IBH was confirmed. Amyand’s hernia occurs when the appendix is present within the inguinal hernia sac, potentially leading to appendicitis. In contrast, an IBH occurs when a portion of the urinary bladder is herniated into the inguinal canal. Although both conditions are rare, they can occur simultaneously. Both hernias can occur in patients of all ages. Amyand’s hernia can result in inflammation, infection, or perforation [4]. Mortality rates of Amyand’s hernia range from 14% to 30%, primarily due to peritoneal sepsis. Other literature reported infection rates as high as 50% [6]. Mortality rates for IBH are not well documented but are generally lower than those for more complex hernias with significant intra-abdominal complications. Although the risk of mortality is low, complications such as urinary retention, bladder dysfunction, incarceration, or strangulation of the bladder can lead to severe outcomes, including sepsis [8].

Cases of definitive preoperative diagnosis of both hernia types are rare. Clinically, Amyand’s hernias and IBH can be challenging to distinguish from other types of inguinal hernias and are often overlooked preoperatively due to symptom overlap with various conditions. The intraoperative diagnosis of IBH is associated with a significant increase in the risk of iatrogenic bladder injury, a complication reported in approximately 12% of cases [9]. Some literature suggests preoperative imaging modalities such as ultrasound or CT can assist in identifying the appendix within the hernia sac or detecting bladder involvement. Both conditions are frequently diagnosed incidentally during surgical intervention; however, others have recommended early surgical intervention as a critical intervention to prevent complications such as bladder dysfunction, appendicitis, or bowel ischemia. The standard surgical approach typically induces hernia repair and appendectomy if appendicitis is present and addresses any bladder involvement. It is not yet standard practice in the surgical field to do routine imaging during the preoperative evaluation of inguinal hernias, particularly when immediate surgical intervention is indicated [10]. Further research of current literature is recommended to determine if the standard of care guidelines should be changed to recommend routine preoperative evaluation of inguinal hernias with CT imaging studies.

## Conclusions

Achieving a definitive preoperative diagnosis of Amyand’s hernia and IBH is clinically challenging due to their nonspecific presentation. Standardized diagnostic and treatment protocols to reduce the risk of intra- or postoperative complications have not yet been established, and these hernias are often only discovered incidentally during surgical procedures. If left undiagnosed, both Amyand’s hernia and IBH can develop life-threatening complications. Although CT imaging is not routinely used as the first-line evaluation for inguinal hernias, early use would facilitate prompt, accurate diagnosis and reduce the risk of misdiagnosis. The limited literature on these uncommon hernias further complicates physicians' ability to become well-versed in their recognition and management. We recommend further research into the balance between the benefits and potential drawbacks of implementing standardized screening and management protocols for rare inguinal hernias, such as Amyand’s hernia and IBH, which can lead to life-threatening complications. While standardization may improve early detection and prevent serious outcomes, it could also place a significant strain on medical resources. For example, incorporating more advanced imaging, such as routine CT scans, into hernia workups would increase healthcare costs and resource utilization. Careful consideration is needed to weigh the benefits of early diagnosis against the financial and logistical impacts on the healthcare system.
